# Influences of *Lactiplantibacillus plantarum* dy-1 Fermentation on the Bitterness of Bitter Melon Juice, the Composition of Saponin Compounds, and Their Bioactivities

**DOI:** 10.3390/foods13203341

**Published:** 2024-10-21

**Authors:** Juan Bai, Zihan Yang, Wei Luo, Ying Zhu, Yansheng Zhao, Beibei Pan, Jiayan Zhang, Lin Zhu, Shiting Huang, Xiang Xiao

**Affiliations:** School of Food and Biological Engineering, Jiangsu University, Zhenjiang 212013, China; 1000005134@ujs.edu.cn (J.B.); yzhyhjx@163.com (Z.Y.); a32315987371524@outlook.com (W.L.); ying307@126.com (Y.Z.); zhaoys@ujs.edu.cn (Y.Z.); 17803780879@163.com (B.P.); jiayanzhang1988@163.com (J.Z.); zhulin19820402@ujs.edu.cn (L.Z.); huangshiting21@126.com (S.H.)

**Keywords:** *Lactiplantibacillus plantarum* dy-1, bitter melon, bitterness, saponins, bioactivities

## Abstract

Lactic acid bacteria fermentation is a beneficial bioprocessing method that can improve the flavor, transform nutrients, and maintain the biological activity of foods. The aim of this study is to investigate the effects of *Lactiplantibacillus plantarum* dy-1 fermentation on the nutritional components, flavor and taste properties, and composition of saponin compounds and their hypolipidemic and antioxidant activities. The results suggested that the total polyphenol content increased, and the soluble polysaccharides and total saponin contents decreased in fermented bitter melon juice (FJ) compared with those in non-fermented bitter melon juice (NFJ). The determination of volatile flavor substances by GC-MS revealed that the response values of acetic acid, n-octanol, sedumol, etc., augmented significantly, and taste analysis with an electronic tongue demonstrated lower bitterness and higher acidity in FJ. Furthermore, UPLC-Q-TOF-MS/MS testing showed a significant decrease in bitter compounds, including momordicines I and II, and a significant increase in the active saponin momordicine U in the fermented bitter melon saponin group (FJBMS). The in vitro assays indicated that FJBMS exhibited similar antioxidant activities as the non-fermented bitter melon saponin group (NFBMS). The in vitro results show that both NFBMS and FJBMS, when used at 50 μg/mL, could significantly reduce fat accumulation and the malondialdehyde (MDA) content and increased the catalase (CAT) activity, while there was no significant difference in the bioactivities of NFBMS and FJBMS. In conclusion, *Lactiplantibacillus plantarum* dy-1 fermentation is an effective means to lower the bitterness value of bitter melon and preserve the well-known bioactivities of its raw materials, which can improve the edibility of bitter melon.

## 1. Introduction

*Momordica charantia* L. belongs to the family Cucurbitaceae and is commonly known as bitter melon or bitter gourd. It is widely grown in tropical and subtropical regions and is rich in a variety of active constituents, such as polysaccharides, saponins, peptides, flavonoids, and alkaloids [1,2]. Many studies have proved that bitter melon and its active ingredients have high medicinal value, with hypoglycemic and hypolipidemic effects [3,4].

However, its food value is greatly reduced due to its bitter taste. All parts of the bitter melon are bitter and the components responsible for this bitter taste are cucurbitane-type triterpenes, such as momordicine II, momordicoside L, momordicine I, and momordicoside K [5,6]. Although it is widely accepted that the bitter taste of bitter melon is related to its functional activities, this relationship has not been critically evaluated [7]. Bitter melon saponins (BMS), which are found in the roots, stems, leaves, and fruits of bitter melon, have the ability to alleviate lipid deposition and offer antioxidant, hypoglycemic and immunomodulatory bioactivities [8,9,10,11]. Through mouse experiments, Tong et al. found that the oral administration of bitter melon saponins could effectively improve lipid metabolism and antioxidant capacity in mice [12]. Deng et al. also found that bitter melon saponins could exert hypoglycemic effects on diabetic mice through multiple pathways [13].

Currently, different processing technologies for fruits and vegetables, including superfine grinding, microbial fermentation, ultrasonic treatments, and so on, are beneficial to the extraction of their active components, improving the nutritional value and biological activity of the substance [14]. Among them, microbial fermentation is a traditional process that uses endogenous or exogenous microorganisms to convert raw materials into products suitable for human needs through specific metabolic pathways. Compared with other processing methods, microbial fermentation has the advantages of low harmful substance usage and low energy consumption [15,16]. In fermented foods, lactic acid bacteria are the most commonly used active agent, and they can improve food flavor, transform nutrients, and enhance bioactivity [17]. Early in 1998, Gupta, U. et al. applied a kind of lactic acid bacteria, *Pediococcus pentosaceus*, in the fermentation of bitter gourds and found that enhanced fat, pyridoxine, and ascorbic acid levels markedly increased [18]. Wen et al. used *Lactiplantibacillus plantarum* NCU116 to ferment bitter melon polysaccharides and found that its usage improved intestinal flora disorders and promoted the growth of beneficial bacteria [19]. Hartajanie et al. found that the fermentation of bitter melon with *Lactobacillus fermentum* LLB3 improved its ability to lower the levels of fasting and postprandial blood glucose [20]. Wang et al. found that the fermentation of *Lactiplantibacillus plantarum* 67-25-2 released more active flavonoid glycosides (isorhamnetin) in sea buckthorn, significantly increasing the total phenolic content and antioxidant activity of sea buckthorn juice [21]. However, there are few studies on bitter melon saponin components and their hypolipidemic and antioxidant activities after fermentation with lactic acid bacteria. Herein, the *Lactiplantibacillus plantarum* dy-1 that we used was previously isolated from pickles and possessed high biological activity levels for fermenting fruit, vegetables, and grains [22]. Our previous studies have elucidated that the extracts of fermented barley, red rice, or other products treated with *Lactiplantibacillus plantarum* dy-1 show high anti-obesity, antitumor, or hypoglycemic effects in cell or animal models [23,24,25].

*Caenorhabditis elegans* (*C. elegans*) has become a common animal model for studies about lipid metabolism or neurodegenerative disease, due to its well-known genetic information, time-saving properties, easy handling, etc. [26]. Moreover, Evans et al. found that these nematodes contain more than 60% to 80% homology with human disease-related genes [27], the results of which could provide a research basis for human studies. A large number of *C. elegans* gene databases and genetic research tools have been established, which can be used to intuitively perform functional analysis [28].

The aim of this study was to investigate the effect of *Lactiplantibacillus plantarum* dy-1 on the flavor and taste of bitter melon juice, the composition of its saponin components, and the bioactivities of saponins in vitro and in vivo, using *C. elegans* as a model. This provides a basis for the production of low-bitterness, high-nutrition functional drinks or dietary supplements using bitter melon as a raw material.

## 2. Materials and Methods

### 2.1. Chemicals and Reagents

The fresh bitter melons used in this research were long green bitter melons from the Weifang Agricultural Station (Weifang, China). The *C. elegans* specimens were gifts from Mr. Wang Dayong’s group at Southeast University. Triglyceride (TG), catalase (CAT), malondialdehyde (MDA), and other biochemical assay kits were purchased from Nanjing Jiancheng Bioengineering Institute (Nanjing, China). The protein concentration determination kit was purchased from Beyotime (Shanghai, China). The rest of the biochemical reagents were purchased from Shanghai Sinopharm Chemical Reagent Co., Ltd. (Shanghai, China).

### 2.2. Preparation of Fermented Bitter Melon Juice and the Determination of Nutritional Component Contents

Fresh bitter melon was crushed with a juicer to produce fresh bitter melon juice (Fresh). Then, a portion of the juice was pasteurized (70 °C, 20 min) to obtain unfermented bitter melon juice (NFJ). The unfermented bitter melon juice was inoculated with 10^6^ CFU/mL *Lactiplantibacillus plantarum* dy-1 and placed in a shaker at 31 °C for 24 h to obtain fermented bitter melon juice (FJ). Finally, 50 g of bitter melon juice was mixed with 1 mL of bacterial solution. In order to favor fermentation, 2% glucose was added to the bitter melon juice in each group. The three kinds of bitter gourd juices were then filtered through eight layers of gauze and the filtrates were taken for further determination. The determination of pH was achieved with a pH meter. The total phenols were extracted using methanol and the content was determined using the Folin–Ciocalteu method, referring to the method of Alias et al. [29]. Soluble polysaccharide content was determined using the phenol sulfate method [30].

### 2.3. Analysis of Flavor Property by Gas Chromatography-Mass Spectrometry

The parameters used herein were based on those described in other studies [31,32]. For the gas chromatography-mass spectrometry (GC-MS) analysis, an accurately weighed amount of 5 mL of bitter melon juice was placed in a 20 mL headspace flask. After equilibration at 40 °C for 1 min, headspace solid-phase microextraction was performed for 20 min.

During the GC analysis, the temperature of the injection port was 250 °C. The extraction head was resolved for 5 min after injection. The carrier gas was of high-purity helium at a flow rate of 1 mL/min. A capillary GC column DB-5 (30 m × 0.25 mm × 0.25 μm) was used. The temperature was maintained at 40 °C for 2 min, increased to 200 °C at 5 °C/min, increased to 240 °C at 10 °C/min, and then maintained for 5 min. MS analysis was performed using an EI ion source. The temperature was 230 °C and the energy was 70 eV. Full-scan mode was used, and the scanning mass number range was 35–450 *m*/*z*.

### 2.4. Electronic Tongue Assay

Samples of bitter melon juice were filtered through a 0.45 μm membrane and determined using the α-ASTREE electronic tongue system (Alpha M.O.S., Toulouse, France). The room temperature was maintained at 25 °C. The data acquisition time was 120 s and the stirring rate was 1 time/s. After the sample was analyzed, the sensor probe was washed with distilled water for 10 s before the next analysis. During the use of the electronic tongue, the temperature was kept stable during the test, with no strong electromagnetic field interference and no strong light irradiation of the instrument. Data were taken 3 times after stabilization and each sample was repeated 7 times.

### 2.5. Extraction of Bitter Melon Saponin and Determination of the Components by UPLC-Q-TOF-MS/MS

To extract the saponins, 100 g of unfiltered bitter melon juice was weighed, and 1 L of 80% ethanol was added. The extract was refluxed twice at 80 °C, then the filtrate was collected and concentrated under reduced pressure. The saponins were then defatted twice with petroleum ether and extracted three times with an equal volume of saturated n-butanol. These n-butanol phases were combined and concentrated under reduced pressure at 70 °C until the liquid was brown and viscous. They were then dissolved with 100 mL of anhydrous methanol, and then 100 mL of acetone was added to produce a precipitate. Then, the mixture was centrifuged at 8000 r/min for 15 min to obtain the crude extract of bitter melon saponin. The crude extract solution of bitter melon saponin was purified using a macroporous resin AB-8 column to obtain bitter melon saponin extract. Fresh bitter melon saponin (FBMS), unfermented bitter melon saponin (NFBMS), and fermented bitter melon saponin (FJBMS) were sequentially extracted from the Fresh, NFJ, and FJ samples. The purified recoveries were above 80%. The determination of saponin content was conducted according to the method developed by Bai, with ginsenoside Rg1 as a standard [33].

BMS samples were filtered using a 0.22 μm microporous filter membrane. A Waters ACQUITY UPLC HSS T3 column (2.1 mm × 100 mm, 1.8 µm) was used for chromatographic analysis. The mobile phases were divided into A (0.1% formic acid-water) and B (0.1% formic acid-acetonitrile). The flow rate was controlled at 0.5 mL/min and the column temperature was 40 °C. The injection volume was 1 μL. The gradient elution procedure was conducted as shown in Table 1.

Mass spectrometry analysis was then performed using a quadrupole time-of-flight mass spectrometer equipped with a Waters electrospray ionization source. The ion source temperature was 100 °C and the samples were detected in positive and negative ion scan mode. The dissolved gas temperature was 450 °C, the cone gas flow rate was 50 L/h, and the dissolved gas flow rate was 900 L/h. The scanning time was 0.2 s and the mass-to-charge ratio scanning range was 50–1500 m/z. The collision energy was 30–50 V for positive ion mode and 40–60 V for negative ion mode. Leucine enkephalin (*m*/*z* 556.2771 ESI^+^, *m*/*z* 554.2615 ESI^-^) was used for mass-axis correction during the process, to ensure the accuracy of the mass spectral data. The liquid-mass system was controlled by the MassLynx 4.1 software, and the data were acquired in Continuum data format.

### 2.6. Determination of Antioxidant Activities In Vitro

ABTS and DPPH scavenging rates were measured in previous publications, and these were used with small modifications [34], using vitamin C (VC) as a reference. First, 20 μL of BMS was added to 180 μL of ABTS solution or methanol solution as the sample group and control group, respectively; 20 μL of distilled water with 180 μL of ABTS solution was taken as the blank group. After leaving the mixture to stand away from any light for 30 min, the absorbance was measured at 740 nm. The clearance of ABTS was calculated as follows:

(1) $$\%Clearance=(1-\frac{A_{sample}-A_{blank}}{A_{control}})\times 100\%$$

where $A_{sample}$, $A_{blank}$ and $A_{control}$ are the absorbance values measured for the sample, blank, and control groups, respectively.

For the DPPH assay, 10 μL of BMS with the addition of either 190 μL of DPPH solution or methanol solution was taken as the sample group and control group, respectively. Furthermore, 10 μL of distilled water plus 190 μL of DPPH solution was taken as the blank group, which was left to stand for 30 min away from any light. The absorbance was measured at 517 nm. DPPH clearance was calculated as follows:

(2) $$\%Clearance=(1-\frac{A_{sample}-A_{blank}}{A_{control}})\times 100\%$$

where $A_{sample}$, $A_{blank},$ and $A_{control}$ are the absorbance measured for the sample, blank, and control groups, respectively.

### 2.7. Effect of Bitter Melon Saponin on the Hypolipidemic and Antioxidant Activities of Nematodes

*C. elegans* were cultured at 20 °C using a nematode growth medium (NGM) containing *Escherichia coli* (*E. coli*) OP50. Working according to Bai et al., the synchronization treatment was carried out using sodium hydroxide with sodium hypochlorite [35]. Then, the synchronization-treated L1 nematodes were transferred to the NGM, divided into a blank control group (Control), 2% glucose model group (model), 2% glucose + fresh bitter melon saponin group (FBMS), 2% glucose + non-fermented bitter melon saponin group (NFBMS), and 2% glucose + fermented bitter melon saponin group (FJBMS), and then cultured at 20 °C. After 48 h of BMS treatment, the nematodes were washed three times with M9 buffer to determine the relevant indexes.

To determine the body length and width, the nematodes were first fixed using 10% isopropyl alcohol. Then, the photographs were taken with an inverted microscope (Nikon Corporation, 120c) and determined with Image J. The observation of oil red O staining of nematodes after 48 h of BMS treatment, using the method developed by Huang et al., required 2 h of immersion in oil red O stain after isopropanol fixation [36]. The nematodes were crushed using ultrasound before determining the triglyceride (TG) and malondialdehyde (MDA) contents and catalase (CAT) activity. Then, the samples were centrifuged at 5000 r/min for 10 min and the supernatant was removed. TG and MDA content and CAT activity were determined according to the requirements of the kit. Triplicate runs were performed for each group.

### 2.8. Statistical Analysis

The results were expressed as mean ± standard deviation (SD) and plotted using GraphPad Prism 9.5. The test data were analyzed for significance between multiple groups using the one-way ANOVA module in SPSS 26.0, with a significance level of 0.05.

## 3. Results and Discussion

### 3.1. Effect of dy-1 Fermentation on the Nutritional Components and Flavor Properties of Bitter Melon Juice

**Figure 1:**
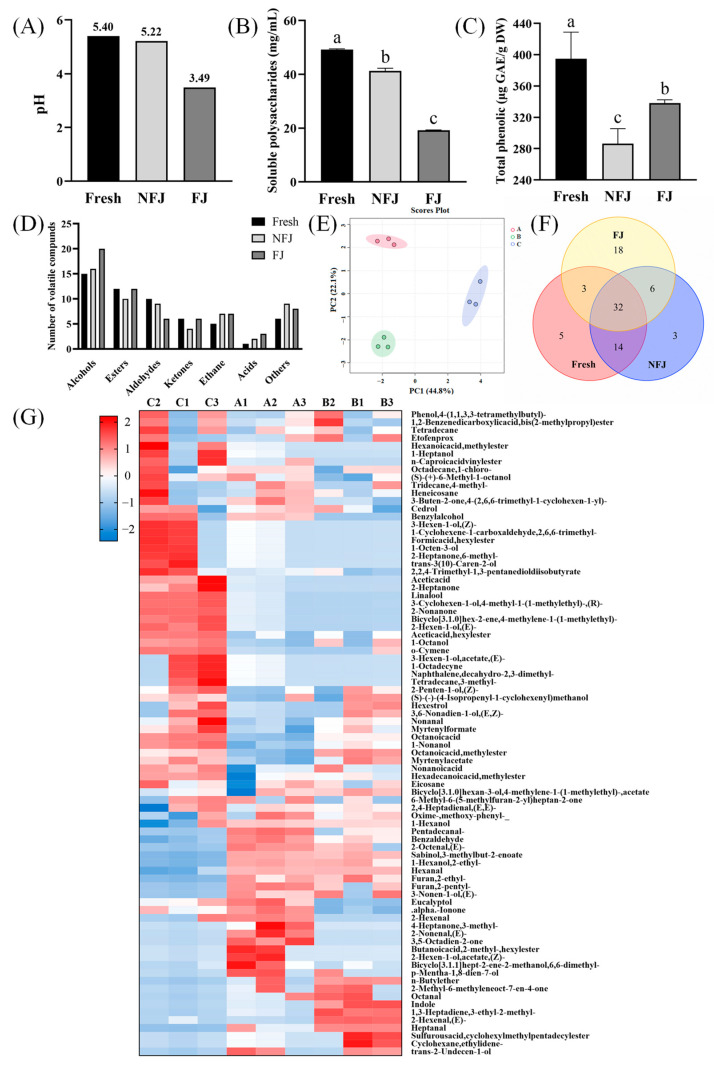
Effect of fermentation on the substance composition of bitter melon juice in each group. (**A**) Determination of the pH of bitter melon juice with a pH meter. (**B**) Determination of soluble polysaccharides in bitter melon juice by the phenol sulfuric acid method. (**C**) Determination of total phenols of bitter melon juice, using the Folin–Ciocalteu method. (**D**–**G**) Substance number plots, PCA plots, Venn diagrams, and heatmap, obtained from the determination of volatile compounds in bitter melon juice by GC-MS. A, B, and C represent Fresh, NFJ, and FJ, respectively. Different letters indicated the variability of the three groups of samples (*p* < 0.05).

Firstly, the changes in pH and sensory indexes of bitter melon juice before and after dy-1 fermentation were determined. As shown in Figure 1A, the pH of bitter melon juice decreased gradually with fermentation time, and the difference between Fresh and NFJ was small. The pH of the bitter melon juice decreased significantly after dy-1 fermentation to only 3.49. Then, the effect of fermentation on the composition of the substances was explored by determining the polysaccharide and polyphenol contents and their volatile components. Polysaccharide is a widely studied bioactive component, with various functional activities such as antioxidant and antiviral properties [37]. As shown in Figure 1B, the soluble polysaccharide content decreased after heating, which might be from the degradation of polysaccharides caused by high temperatures [38]. After dy-1 fermentation, the soluble polysaccharide content decreased further. Wang et al. obtained similar results, largely due to polysaccharide utilization by microorganisms during fermentation [39].

Polyphenols are active components with pharmacological effects, such as antioxidant and anticancer properties. Measurement of the total phenol content provides valuable information for assessing the impact of dietary interventions [40]. As shown in Figure 1C, compared with unfermented bitter melon juice, the total phenol content increased significantly after dy-1 fermentation, which finding was in agreement with the study by Gao et al. [41]. It was largely due to the production of some phenolic compounds by microorganisms through secondary metabolic pathways during the fermentation process [42].

In order to clarify the effect of fermentation on the flavor properties, GC-MS analysis was applied to determine the volatile components. The chromatograms of three kinds of bitter melon juice are shown in Figure S1. As shown in Table 2, a total of 81 volatile flavor compounds were detected, including 25 alcohols, 15 esters, and 11 aldehydes, as well as some acids and other heterocyclic compounds. The number of alcohols, esters, and acids increased, while the number of aldehydes decreased in FJ compared to NFJ (Figure 1D). Lu et al. demonstrated that exogenous *Lactiplantibacillus plantarum* tended to convert aldehydes to alcohols [43]. The PCA plots show that the volatile compositions of the three groups were markedly different (Figure 1E); among them, 32 common substances were present in all three kinds of bitter melon juices (Figure 1F).

As shown in Figure 1G, the characteristic volatile substances of Fresh and NFJ were more similar, after dy-1 fermentation which were different from those in FJ. In Fresh, alcohols and aldehydes were predominant, containing 41.71% and 40.95%, respectively, while, the content of alcohols decreased to 35.72% and aldehydes increased to 47.12% after heating and sterilization. However, after dy-1 fermentation, alcohols predominated at 45.70% in the FJ, and aldehydes were strikingly reduced to 17.04%. As the odor thresholds of alcohols are relatively high, they showed little effect on the characteristic odor of fermented vegetables, even though alcohols present a pleasant aroma. In contrast, most of the aldehydes have low odor thresholds, making a more important impact on the flavor formation of fermented vegetables, which explains the lower level of flavor in the fermented products [44]. Meanwhile, the content of esters increased significantly from 7.45% to 27.81% during fermentation, in which hexyl acetate and hexyl formate were elevated the most. Pan et al. found that hexyl acetate possessed apple and herbaceous aromas, and hexyl formate had an apple and unripe plum-like sweet aroma [45]. Both of them exhibited flavor-modifying effects on fermented bitter melon juice in the present study. The fermentation also produced a new organic acid, acetic acid, which is in agreement with the study by Chen et al. [46]. Acetic acid could aggravate the sourness of fermented bitter melon juice by giving an acid odor, and could also lead to a significant decrease in the pH of fermented bitter melon juice. Above all, fermentation increased the total phenolic content, decreased the soluble polysaccharide content, and altered the composition of the volatile compounds in bitter melon juice.

### 3.2. Changes in the Taste Fingerprints of Fermented Bitter Melon Juice

**Figure 2:**
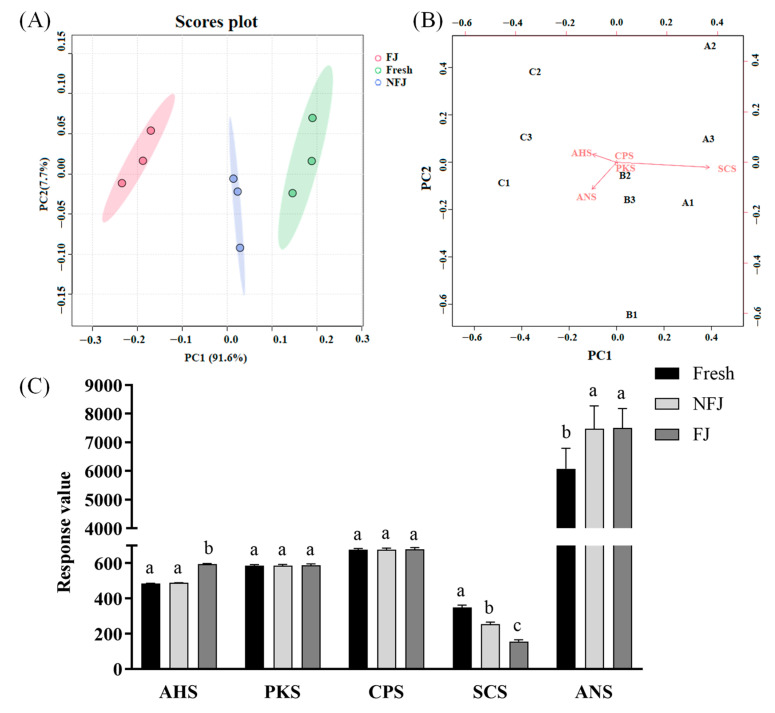
Effects of fermentation on the taste of bitter melon juice. (**A**) and (**B**) Principal component analysis of three kinds of bitter melon juice, based on the results from electronic tongue determination. As shown in Figure (**B**), A, B, and C, respectively, represent Fresh, NFJ, and FJ. (**C**) The sensor data of bitter melon juice was determined by the electronic tongue, and is divided into AHS (acidity), PKS (general purpose), CPS (general purpose), SCS (bitterness), and ANS (sweetness). Different letters indicate the variability of the three groups of samples (*p* < 0.05).

The electronic tongue is a highly sensitive and efficient instrument for taste analysis, which can classify and identify samples of different compositions, based on an array of taste sensors [47,48]. The results of PCA analyses are shown in Figure 2A, in which electronic tongue detection could completely distinguish between the tastes of the three groups of bitter melon juice. Moreover, the greatest contribution to the differences was the bitter taste, followed by the indicators of sourness and sweetness (Figure 2B), which indicated that the main difference in taste among the three kinds of bitter melon juices lay in the bitter taste. Figure 2C shows the results of the electronic tongue determination of bitter melon juice using various probes for AHS (acidity), PKS (general purpose), CPS (general purpose), SCS (bitterness), and ANS (sweetness). It can be observed that there was an obvious difference in the bitter taste of the bitter melon juice. Fresh had the highest bitter taste response value, followed by NFJ, and FJ had the least bitter response value, suggesting a striking reduction in bitterness after dy-1 fermentation. Mazlan et al. also found that fermentation by *Lactiplantibacillus plantarum* BET003 was able to decrease the bitter taste of bitter melon, which is largely due to the lower saponin content [49]. In addition, the acidity response value of FJ was significantly higher than those of the other two groups, which is consistent with the changes in pH value. Regarding the augmented sweetness in NFJ and FJ, this could be ascribed to high-temperature sterilization, which increased the reducing sugar content.

### 3.3. Effect of dy-1 Fermentation on the Composition of Bitter Melon Saponin

**Figure 3:**
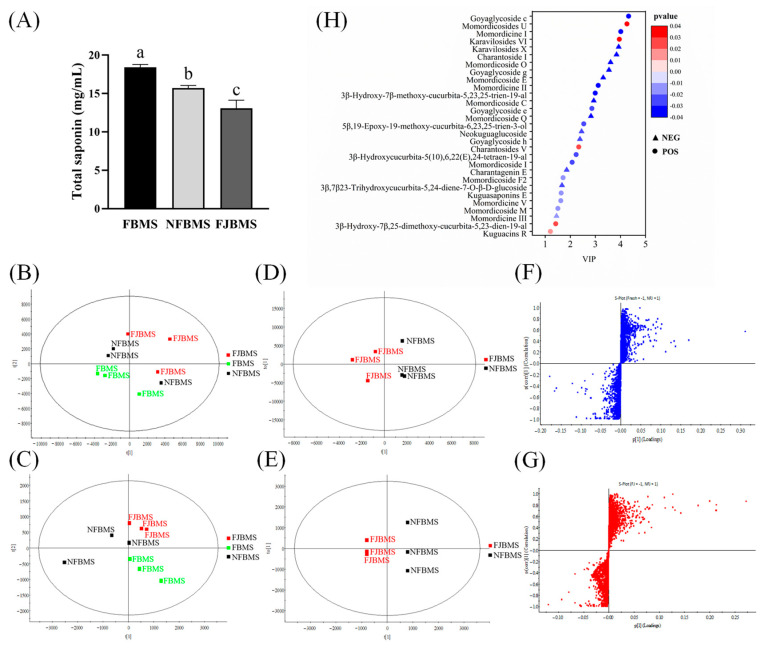
Effect of fermentation on the saponin composition of bitter melon juice in each group. UPLC-Q-TOF-MS/MS was used to determine the saponin composition of each group. (**A**) Determination of the total saponin of BMS. (**B**,**C**) PLS-DA score plots of three kinds of bitter melon juices in positive and negative ion modes. (**D**,**E**) Positive and negative ion OPLS-DA score plots of unfermented and fermented bitter melon samples. (**F**,**G**) Positive and negative ion S-plot graphs of unfermented and fermented bitter melon samples. (**H**) Significant changes in composition before and after dy-1 fermentation. Different letters indicate the variability of the three groups of samples (*p* < 0.05).

As the saponins in bitter melon are the main source of its bitterness, we speculated that the changes in saponins resulted in a reduction in the bitterness of bitter melon juice [50]. Herein, we further extracted the saponins from three kinds of bitter melon juice to investigate the effect of dy-1 fermentation on saponin contents and composition. As shown in Figure 3A, after sterilization, the saponin concentration significantly decreased in NFJ and FJ (named NFBMS and FJBMS, respectively) compared to that in Fresh bitter melon juice (FBMS), greatly due to the degradation of saponins by high temperatures. Moreover, the content of FJBMS was lower than in NFBMS, demonstrating that dy-1 fermentation enhanced the biotransformation of saponins. In agreement with our results, Gao et al. also found that the saponins in sterilized and fermented juices significantly decreased, on account of the enzyme activities of *Lactiplantibacillus plantarum* (such as β-glucosidase) which were responsible for hydrolyzing momordicoside to aglycones [41].

Therefore, we next applied UPLC-Q-TOF-MS/MS analysis to evaluate the changes in the composition of bitter melon saponins before and after dy-1 fermentation. BPI plots of the positive and negative ions are shown in Figures S2–S4. Figure 3B,C shows the PLS-DA scores of the three kinds of bitter melon saponin samples in positive and negative ion modes, respectively, indicating that FBMS, NFBMS, and FJBMS samples could be clearly distinguished due to the significant differences in the saponin composition. Then, the saponin compositions of NFBMS and FJBMS were further compared. As shown in the OPLS-DA score plot (Figure 3D,E), NFBMS and FJBMS were significantly divided into two groups in positive and negative ion modes. Furthermore, the S-plot (Figure 3F,G) revealed that there were some key saponins that were scattered around the edges, which contributed to the discrimination of NFBMS and FJBMS. Based on the above discriminatory results, the variable importance projection index of VIP > 1 and *p* < 0.05 difference compounds was selected between NFBMS and FJBMS. Either 17 or 12 kinds of saponin components were detected in the positive or negative ion mode, as shown in Table 3. It is known that momordicoside K, momordicoside L, momordicine I, and momordicine II are the most important bitter saponin components of bitter melon, in which momordicine I and momordicine II could be detected in positive ion mode in this study [51]. We also conducted a VIP plot analysis in positive ion mode to illustrate the variables. As shown in Figure 3H, the contents of goyaglycoside c, momordicines I and II, etc., were significantly lower, and momordicine U was significantly higher in FJBMS compared to NFBMS. Among them, momordicine I and II were two identified bitter triterpenoids, which had the greatest influence on the bitterness of bitter melon. In addition, Deng et al. determined that goyaglycoside c was more bitter than caffeine, which was equal to the equivalent of 4.32 mg of caffeine per milligram of bitterness value [7]. The reduced content of goyaglycoside c and momordicine I and II were responsible for the reduced bitter taste of bitter melon juice after dy-1 fermentation. Most important of all, we found that momordicine U remarkably increased more than twofold after dy-1 fermentation; this is an active monodesmoside cucurbitane and could be deglycosylated to be momordicine I [52]. As the aglycone of momordicine U, momordicine I shares the same basal skeleton. It has been reported that momordicine I has shown less bioactivity in stimulating insulin secretion than momordicine U, due to the fact that glycosylation most likely accounts for the lower bioactivity [53,54]. Thus, we theorized that there must be some specific enzymes during dy-1 fermentation that play a great role in the biotransformation of saponins, which needed further confirmation. Consequently, it could be seen that *Lactiplantibacillus plantarum* dy-1 fermentation was an effective method to debitter bitter melon juice via bioconversion.

### 3.4. Effect of dy-1 Fermentation on the Antioxidant Activity of BMS In Vitro

**Figure 4:**
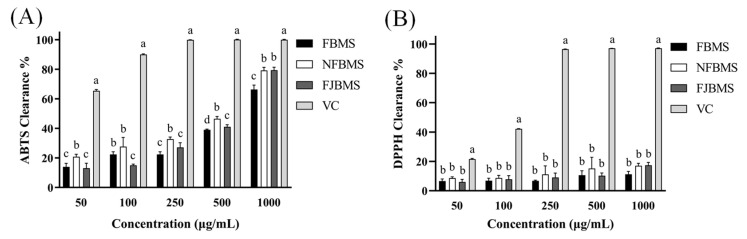
Effect of fermentation on the antioxidant activity of BMS in vitro. (**A**) ABTS and (**B**) DPPH clearance of BMS and VC at different concentrations. Different letters indicate the variability of the four groups of samples at each concentration (*p* < 0.05).

In the follow-up experiments, we also identified how dy-1 fermentation influenced the antioxidant activities of bitter melon saponins. The measurements of ABTS and DPPH radical clearance represent two common indicators of antioxidant activity [55]. As shown in Figure 4, the ABTS and DPPH clearance of the four groups showed an increasing trend with increasing concentration. The clearance of VC was significantly higher than that of BMS, indicating the higher antioxidant activity of VC. Among the three kinds of BMS, the NFBMS showed a higher clearance of ABTS than the other two groups at all concentrations except 1000 μg/mL, at which point the ABTS clearance of NFBMS was similar to that of FJBMS. As for the clearance of DPPH, there was no significant difference among the three kinds of BMS, and the percentages were greatly lower than that of VC, which might be related to the relatively low content of polyphenols [34].

It can be seen that dy-1 fermentation not only reduced the saponin contents and changed the composition of BMS but also decreased the antioxidant activities of BMS at the same concentration in vitro. Chen et al. found that different fermentation conditions may lead to the production of different types of saponins, which, in turn, lead to significant differences in their antioxidant properties [56]. Similar results were found by Sanaz Arjmand et al., uncovering that *Lactiplantibacillus plantarum* fermentation reduced the saponin content of quinoa dough and slightly decreased the antioxidant capacity after 12 h of fermentation, which can be related to the reduction in antioxidant compounds such as saponins and total phenolics [57].

### 3.5. Impacts of BMS on Lipid Deposition and Antioxidant Activity of C. elegans In Vivo

**Figure 5:**
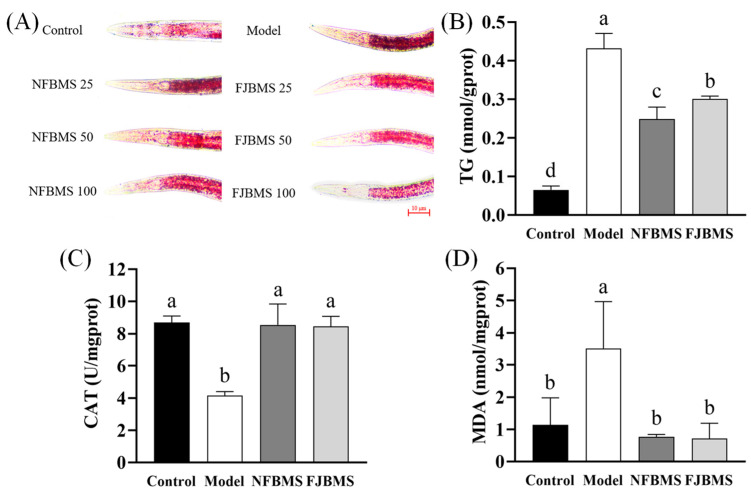
Effect of fermentation on the lipid-lowering and antioxidant activity of BMS. (**A**) L1-stage *C. elegans* nematodes were treated with different concentrations of NFBMS and FJBMS for 48 h. The lipids in the nematodes were stained using oil red O stain. The number following the name represents the administered concentrations. The unit was μg/mL. For example, ‘NFBMS 25’ indicates that NFBMS was administered at a concentration of 25 μg/mL. (**B**–**D**) The TG content, CAT activity, and MDA content of L1 stage *C. elegans* after 48 h of treatment were determined using assay kits. Different letters indicate the variability of the four groups (*p* < 0.05).

In order to find out whether fermentation changes the bioactivities of BMS in vivo, a high-fat model was constructed using *C. elegans* for further study. Working according to our previous study, NFBMS and FJBMS were selected at 25, 50, and 100 μg/mL in the current study [58]. As shown in Table 4, the body length and width of nematodes in the NFBMS and FJBMS groups were not significantly different from the model group at 50 μg/mL, while BMS at 25 and 100 μg/mL obviously inhibited the growth of nematodes. Then, we preliminarily investigated the effect of different concentrations of BMS on lipid deposition in nematodes using oil red O staining. Oil red O staining can visualize the lipid content in nematodes and is one of the most intuitive indicators of lipid-lowering activity [36]. As shown in Figure 5A, the overall fat in nematodes was significantly reduced after treatment with BMS, compared with the model group. Additionally, the difference between 50 μg/mL and 100 μg/mL was not obvious. Above all, 50 μg/mL was chosen as the concentration for subsequent experiments. Moreover, 50 μg/mL of FJBMS exhibited less fat than that of NFBMS, indicating that FJBMS could maintain the lipid-lowering effects as NFBMS did.

Triglyceride is the important endpoint during fat synthesis, and its content directly reflects lipid deposition in the body [59]. During lipid oxidation, a variety of lipid peroxides are formed, such as aldehyde groups (MDA), hydroxyl groups, hydroperoxides, and new oxygen radicals [60]. Oxygen radicals cause cellular damage, not only through the peroxidation of polyunsaturated fatty acids in biological membranes but also from the breakdown products of lipohydroperoxides. As an antioxidant enzyme, CAT can prevent the production of highly toxic hydroxyl radicals and protect organisms from oxidative stress [61]. The measurement of MDA, a lipid peroxidation product, also reflects the degree of lipid peroxidation and the degree of cellular damage in the organism [62]. The higher the level of MDA, the more severe the oxidative damage.

The results revealed that both NFBMS and FJBMS could significantly suppress fat deposition compared to the model group (Figure 5B, *p* < 0.05), in which NFBMS exhibited better activity than FJBMS, indicating that dy-1 fermentation weakened the lipid-lowering effect by changing the composition of BMS. Inconsistent with our data, Shen et al. found that fermentation with *Lactiplantibacillus plantarum* MB11 was able to increase the content of minor ginsenosides and enhance the anticolorectal cancer activity of ginsenosides [63]. Thus, it was indicated that dy-1 fermentation did not increase compounds with high lipid-lowering bioactivity too much, even though momordicosides U was augmented more than twofold. Furthermore, CAT activity was increased strikingly in the BMS-treated groups, and there was no significant difference in CAT activity between NFBMS and FJBMS, indicating that BMS could effectively reduce the hydroxyl radicals induced by obesity. In addition, MDA content was significantly higher in the model group compared to the Control group, while BMS treatment resulted in a substantial decrease without differences between NFBMS and FJBM, suggesting an inhibitory effect on lipid oxidation. Yan et al. explored the effect of fermented rice buckwheat on lipid deposition and antioxidant activities in *C. elegans*, which yielded results similar to our findings [64]. In all, dy-1 fermentation could not only debitter the bitter melon juice by reducing the total saponin contents but also maintain the lipid-lowering and antioxidant activities of saponins.

However, there were still some limitations in the present study. For instance, we did not evaluate the effects of different fermentation conditions, such as temperature and fermentation time, on the bitterness and saponin composition of bitter melon juice, which could affect the bioactivity and chemical composition of the final fermentation product. In addition, dy-1 fermentation increased the sour taste of bitter melon juice while reducing the bitter taste, which could be followed up by optimizing fermentation conditions to further improve the taste and nutritional value of the product.

## 4. Conclusions

In conclusion, the composition of the nutritional components in bitter melon juice was changed during *Lactiplantibacillus plantarum* dy-1 fermentation, as characterized by an increase in total phenol content and a decrease in soluble sugar and saponin contents. Meanwhile, dy-1 fermentation could significantly change the flavor and taste of bitter melon juice by altering the volatile compounds, including alcohols and aldehydes, thereby reducing the bitterness and increasing the acidity. Furthermore, the decreased bitter saponin compounds, especially goyaglycoside c and momordicine I and II, were responsible for the reduced bitterness of the bitter melon juice. Based on the changed composition of bitter melon saponins, we found that dy-1 fermentation did not significantly weaken the bioactivities of saponins, both in vitro and in vivo. Therefore, *Lactiplantibacillus plantarum* fermentation can be regarded as an effective way to debitter bitter melon while maintaining its bioactive properties, thereby improving its value and application in food and dietary supplement products.

## Figures and Tables

**Table 1:** Chromatographic elution procedure.

Time (min)	A (v%)	B (v%)
0	95	5
19	10	90
19.5	2	98
22	2	98

**Table 2:** Flavor composition of bitter melon juice.

Name	Molecular Formula	CAS	Relative Peak Area		
			Fresh	NFJ	FJ
Alcohol compounds					
(S)-(-)-(4-Isopropenyl-1-cyclohexenyl)methanol	C_10_H_16_O	18457-55-1	1,147,228	1,297,133	1,007,856
(S)-(+)-6-Methyl-1-octanol	C_9_H_20_O	110453-78-6	229,925	210,198	342,104
1-Heptanol	C_7_H_16_O	111-70-6	ND	ND	11,586,219
1-Hexanol	C_6_H_14_O	111-27-3	63,135,465	58,161,649	115,615,727
1-Hexanol, 2-ethyl-	C_8_H_18_O	104-76-7	10,511,214	10,724,622	ND
1-Nonanol	C_9_H_20_O	143-8-8	1,046,117	2,418,360	5,024,659
1-Octanol	C_8_H_18_O	111-87-5	ND	3,914,244	6,844,987
1-Octen-3-ol	C_8_H_16_O	3391-86-4	ND	ND	8,888,366
2-Hexen-1-ol, (E)-	C_6_H_12_O	928-95-0	ND	ND	29,894,153
2-Penten-1-ol, (Z)-	C_5_H_10_O	1576-95-0	2,160,168	4,505,637	8,678,142
3,6-Nonadien-1-ol, (E,Z)-	C_9_H_16_O	56805-23-3	ND	1,093,342	1,872,701
3-Cyclohexen-1-ol, 4-methyl-1-(1-methylethyl)-, (R)-	C_10_H_18_O	20126-76-5	ND	ND	3,733,420
3-Hexen-1-ol, (Z)-	C_6_H_12_O	928-96-1	ND	ND	126,966,465
3-Hexen-1-ol, acetate, (E)-	C_8_H_14_O_2_	3681-82-1	ND	ND	2,765,084
3-Nonen-1-ol, (E)-	C_9_H_18_O	10340-23-5	793,771	2,019,006	ND
Benzyl alcohol	C_7_H_8_O	100-51-6	1,215,308	ND	4,308,491
Bicyclo[3.1.0]hexan-3-ol, 4-methylene-1-(1-methylethyl)-, acetate	C_12_H_18_O_2_	3536-54-7	1,121,782	1,344,819	968,306
Bicyclo[3.1.1]hept-2-ene-2-methanol, 6,6-dimethyl-	C_10_H_16_O	515-00-4	96,505,762	83,830,076	90,598,471
Cedrol	C_15_H_26_O	77-53-2	731,266	996,897	2,063,601
Eucalyptol	C_10_H_18_O	470-82-6	10,009,126	7,189,491	11,564,944
Sabinol, 3-methylbut-2-enoate	C_15_H_22_O_2_		4,992,733	5,623,567	ND
Linalool	C_10_H_18_O	78-70-6	ND	ND	10,736,050
trans-2-Undecen-1-ol	C_11_H_22_O	75039-84-8	473,164	489,884	ND
trans-3(10)-Caren-2-ol	C_10_H_18_O	1637413-80-9	ND	ND	626,373
p-Mentha-1,8-dien-7-ol	C_10_H_16_O	536-59-4	894,380	785,207	ND
Ester compounds					
2,2,4-Trimethyl-1,3-pentanediol diisobutyrate	C_16_H_30_O_4_	6846-50-0	547,484	581,317	2,067,736
1,2-Benzenedicarboxylic acid, bis(2-methylpropyl) ester	C_16_H_22_O_4_	84-69-5	491,766	993,310	1,074,061
2-Hexen-1-ol, acetate, (Z)-	C_8_H_14_O_2_	56922-75-9	1,347,906	ND	ND
Hexadecanoic acid, methyl ester	C_17_H_34_O_2_	112-39-0	144,205	149,986	326,174
Hexanoic acid, methyl ester	C_7_H_14_O_2_	106-70-7	ND	ND	1,105,448
Myrtenyl acetate	C_12_H_18_O_2_	1079-1-2	228,471	687,739	815,421
Myrtenyl formate	C_11_H_16_O_2_	72928-52-0	426,441	693,866	976,437
n-Caproic acid vinyl ester	C_8_H_14_O_2_	3050-69-9	4,091,370	ND	13,036,098
Octanoic acid, methyl ester	C_9_H_18_O_2_	111-11-5	ND	1,237,273	867,470
Oxime-, methoxy-phenyl-_	C_8_H_9_NO_2_		29,555,110	31,869,695	29,907,716
Sulfurous acid, cyclohexylmethyl pentadecyl ester	C_22_H_44_O_3_S		ND	300,911	ND
Etofenprox	C_25_H_28_O_3_	80844-7-1	797,696	1,389,211	1,589,981
Acetic acid, hexyl ester	C_8_H_16_O_2_	142-92-7	404,362	592,894	5,330,228
Butanoic acid, 2-methyl-, hexyl ester	C_11_H_22_O_2_	10032-15-2	278,634	ND	ND
Formic acid, hexyl ester	C_7_H_14_O_2_	629-33-4	1,615,549	ND	213,142,427
Aldehyde compounds					
2,4-Heptadienal, (E,E)-	C_7_H_10_O	4313/3/5	3,620,376	4,434,243	6,038,324
2-Hexenal	C_6_H_10_O	505-57-7	93,756,704	127,167,028	138,659,097
1-Cyclohexene-1-carboxaldehyde, 2,6,6-trimethyl-	C_10_H_16_O	432-25-7	ND	ND	5,626,347
2-Nonenal, (E)-	C_9_H_16_O	18829-56-6	427,545	ND	ND
2-Octenal, (E)-	C_8_H_14_O	2548-87-0	2,871,187	2,200,733	ND
Benzaldehyde	C_7_H_6_O	100-52-7	11,650,037	10,084,714	6,164,773
Heptanal	C7H_14_O	111-71-7	674,198	3,855,655	ND
Octanal	C_8_H_16_O	124-13-0	1,798,563	2,501,411	ND
Hexanal	C_6_H_12_O	66-25-1	73,099,577	88,763,450	3,149,155
Nonanal	C_9_H_18_O	124-19-6	2,703,002	4,283,748	5,969,898
Pentadecanal-	C_15_H_30_O	2765-11-9	822,804	215,302	ND
Ketone compounds					
.alpha.-Ionone	C_13_H_20_O	127-41-3	6,013,974	3,815,375	6,486,451
2-Heptanone	C_7_H_14_O	110-43-0	ND	ND	483,217
2-Heptanone, 6-methyl-	C_8_H_16_O	928-68-7	ND	ND	1,157,799
2-Methyl-6-methyleneoct-7-en-4-one	C_10_H_16_O	19860-68-5	851,911	1,026,360	ND
2-Nonanone	C_9_H_18_O	821-55-6	ND	ND	4,092,844
3-Buten-2-one, 4-(2,6,6-trimethyl-1-cyclohexen-1-yl)-	C_13_H_20_O	14901-7-6	3,041,402	2,372,728	3,578,358
3,5-Octadien-2-one	C_8_H_12_O	38284-27-4	4,788,567	ND	ND
4-Heptanone, 3-methyl-	C_8_H_16_O	15726-15-5	624,552	ND	ND
6-Methyl-6-(5-methylfuran-2-yl)heptan-2-one	C_13_H_20_O_2_	50464-95-4	860,231	440,608	1,187,635
Alkane compounds					
Cyclohexane, ethylidene-	C_8_H_14_	1003-64-1	ND	1,689,904	ND
Eicosane	C_20_H_42_	112-95-8	907,845	595,969	1,289,121
Heneicosane	C_21_H_44_	629-94-7	2,935,580	2,241,068	5,405,558
Hexestrol	C_18_H_22_O_2_	5635-50-7	ND	485,921	662,672
Octadecane, 1-chloro-	C_18_H_37_Cl	3386-33-2	999,706	1,233,659	2,846,363
Tetradecane	C_14_H_30_	629-59-4	3,118,315	3,763,902	5,230,837
Tetradecane, 3-methyl-	C_15_H_32_	18435-22-8	ND	ND	302,262
Tridecane, 4-methyl-	C_14_H_30_	26730-12-1	508,934	649,865	1,539,307
Heterocyclic compounds					
Indole	C_8_H_7_N	120-72-9	260,369	780,387	ND
Furan, 2-ethyl-	C_6_H_8_O	3208-16-0	11,781,024	15,908,948	ND
Furan, 2-pentyl-	C_9_H_14_O	3777-69-3	3,041,652	1,913,630	ND
Naphthalene, decahydro-2,3-dimethyl-	C_12_H_22_	1008-80-6	ND	ND	2,789,222
o-Cymene	C_10_H_14_	527-84-4	ND	6,236,597	18,356,075
Phenol, 4-(1,1,3,3-tetramethylbutyl)-	C_14_H_22_O	140-66-9	224,698	554,285	915,276
Acid compounds					
Nonanoic acid	C_9_H_18_O_2_	112-5-0	1,155,186	2,256,306	4,083,797
Acetic acid	C_2_H_4_O_2_	64-19-7	ND	ND	10,285,024
Octanoic acid	C_8_H_16_O_2_	124-07-2	ND	3,866,656	12,813,198
Others					
1,3-Heptadiene, 3-ethyl-2-methyl-	C_10_H_18_	61142-35-6	ND	341,522	ND
Bicyclo[3.1.0]hex-2-ene, 4-methylene-1-(1-methylethyl)-	C_10_H_14_	36262-09-6	ND	ND	5,341,464
1-Octadecyne	C_18_H_34_	629-89-0	ND	ND	2,912,730
n-Butyl ether	C_8_H_18_O	142-96-1	7,070,999	6,056,341	ND

Note: ND meant not detected.

**Table 3:** Saponin constituents, which are significantly different in NFBMS and FJBMS.

NO.	Time (min)	Component Name	Neutral Mass (Da)	Formula	VIP	*p*	Normalized Abundance		Mode	MS/MS	Adducts
							NFJ	FJ			
1	6.37	Momordicoside M	797.47	C_42_H_68_O_14_	1.51	0.02	19,369.64	15,071.19	POS	639.3898; 455.3525; 439.3558; 391.3394	+H, +Na
2	8.63	Goyaglycoside e	781.48	C_42_H_68_O_13_	2.86	0.03	7575.65	1360.32	POS	657.4031; 623.4006; 439.3558; 391.3394	+H, +Na
3	10.58	Momordicine II	657.40	C_36_H_58_O_9_	3.11	0.04	79,318.04	62,066.33	POS	639.3898; 437.3442; 419.3315; 409.3449; 391.3394	+H, +Na
4	10.82	Momordicine V	743.40	C_39_H_60_O_12_	1.62	0.02	3336.70	1104.61	POS	657.4031; 477.3315; 391.3394	+Na
5	11.47	Momordicoside F2	619.42	C_36_H_58_O_8_	1.71	0.02	39,206.78	31,185.88	POS	439.3558; 421.3519; 391.3394; 357.2815	+H, +Na
6	11.7	Momordicoside I	619.42	C_36_H_58_O_8_	2.07	0.02	150,646.85	135,430.89	POS	439.3558; 421.3519; 391.3394; 357.2815	+H, +Na
7	12.21	Momordicine I	495.35	C_30_H_48_O_4_	4.01	0.04	20,872.71	6116.94	POS	477.3391; 437.3442; 419.3315; 391.3394	+Na
8	12.43	Momordicoside C	617.41	C_36_H_56_O_8_	2.94	0.03	76,018.86	58,005.11	POS	599.3913; 477.3391; 391.3394; 321.2568	+H, +Na
9	12.78	Momordicosides U	685.43	C_38_H_62_O_9_	4.26	−0.04	8432.61	25,378.54	POS	639.3898; 419.3315; 391.3394; 309.2590	+Na
10	12.86	Kuguasaponins E	685.43	C_38_H_62_O_9_	1.64	0.02	14,041.64	8655.58	POS	639.3898; 419.3315; 391.3394; 309.2590	+Na
11	13.15	Goyaglycoside c	685.43	C_38_H_62_O_9_	4.32	0.05	49,159.25	27,596.93	POS	639.3898; 419.3315; 391.3394; 309.2590	+Na
12	13.3	3β-Hydroxycucurbita-5(10),6,22(E),24-tetraen-19-al	437.34	C_30_H_44_O_2_	2.23	0.02	8102.54	3526.96	POS	419.3315; 391.3464; 409.3520; 401.3238	+H
13	13.34	Karavilosides VI	669.40	C_37_H_58_O_9_	3.95	−0.05	4444.10	18,510.22	POS	637.3712; 581.3309; 495.3455	+Na
14	14.53	Kuguacins R	495.35	C_30_H_48_O_4_	1.21	−0.01	24,126.64	29,710.06	POS	477.3391; 419.3315; 391.3394	+Na
15	15.74	Charantosides V	647.45	C_38_H_62_O_8_	2.34	−0.02	36,202.87	48,974.45	POS	605.4295; 561.4100; 495.3455; 391.3394	+H, +Na
16	16.54	5β,19-Epoxy-19-methoxy-cucurbita-6,23,25-trien-3-ol	523.38	C_32_H_52_O_4_	2.54	0.02	13,908.65	5344.79	POS	487.3645; 443.3338; 391.3394; 368.4236	+Na
17	17.51	3β-Hydroxy-7β,25-dimethoxy-cucurbita-5,23-dien-19-al	457.37	C_30_H_48_O_3_	1.42	−0.02	34,561.75	37,342.92	POS	439.3632; 391.3394; 309.2590; 213.1694	+H, +Na
18	6.37	Karavilosides X	841.45	C_42_H_68_O_14_	3.93	0.05	15,786.80	9138.98	NEG	634.3300; 615.3900	+HCOO, -H
19	6.5	Goyaglycoside h	859.47	C_42_H_70_O_15_	2.39	0.03	8099.47	5182.29	NEG	813.4450; 651.4076; 633.3993; 489.3665	+HCOO, -H
20	6.8	Neokuguaglucoside	839.44	C_42_H_66_O_14_	2.45	0.03	4768.03	2139.17	NEG	752.3946; 146.9631	+HCOO, -H
21	7.82	Momordicoside Q	697.41	C_36_H_60_O_10_	2.82	0.03	12,368.13	8916.62	NEG	651.4076; 401.0886; 325.2057; 307.1886	+HCOO, -H
22	9	Momordicoside O	871.47	C_43_H_70_O_15_	3.61	0.04	5587.93	151.80	NEG	691.4090; 633.3904	+HCOO
23	9.41	Goyaglycoside g	855.47	C_43_H_70_O_14_	3.54	0.04	5073.53	160.79	NEG	617.3918; 513.3471; 202.9929	+HCOO
24	9.53	Momordicoside E	695.40	C_37_H_60_O_12_	3.31	0.04	3882.96	172.67	NEG	513.3312; 314.9821; 146.9631	-H
25	9.95	Charantoside I	675.41	C_37_H_58_O_8_	3.84	0.05	5787.02	1533.38	NEG	633.4258; 405.3022; 352.9889	+HCOO
26	10.34	Momordicine III	647.38	C_36_H_56_O_10_	1.45	0.02	619.58	62.18	NEG	326.9860; 264.9877; 240.9861	-H
27	10.86	3β,7β23-Trihydroxycucurbita-5,24-diene-7-O-β-D-glucoside	665.42	C_36_H_60_O_8_	1.67	0.02	3308.72	2433.39	NEG	633.3904; 587.3901; 146.9631	+HCOO
28	12.88	Charantagenin E	707.43	C_38_H_62_O_9_	1.86	0.02	11,909.27	10,058.02	NEG	376.9863; 276.9907; 255.2324	+HCOO
29	13.42	3β-Hydroxy-7β-methoxy-cucurbita-5,23,25-trien-19-al	513.36	C_31_H_48_O_3_	2.99	0.04	4061.34	1551.21	NEG	376.9863; 314.9821; 264.9877; 255.2324	+HCOO, -H

**Table 4:** Effect of different concentrations of NFBMS and FJBMS on the body length and width of *C. elegans*.

Group	Length (μm)	Width (μm)
Control	742.56 ± 23.94 ^ab^	44.46 ± 2.98 ^abc^
Model	762.89 ± 67.58 ^a^	51.08 ± 10.22 ^ab^
NFBMS 25	651.24 ± 16.46 ^c^	36.69 ± 2.05 ^c^
NFBMS 50	721.37 ± 34.74 ^abc^	53.15 ± 6.51 ^a^
NFBMS 100	682.27 ± 15.57 ^bc^	43.62 ± 2.01 ^abc^
FJBMS 25	676.42 ± 31.07 ^bc^	41.12 ± 3.05 ^b^
FJBMS 50	785.22 ± 49.19 ^a^	45.93 ± 7.41 ^abc^
FJBMS 100	457.33 ± 42.27 ^d^	24.48 ± 1.26 ^d^

Note: The number following the name in the groups represents the administered concentrations. The unit was μg/mL. For example, ‘NFBMS 25’ indicates that NFBMS was administered at a concentration of 25 μg/mL. Different letters indicate the variability of the groups (*p* < 0.05).

## Data Availability

The original contributions presented in the study are included in the article/Supplementary Material, further inquiries can be directed to the corresponding author.

## Supplementary Materials

The following supporting information can be downloaded at: https://www.mdpi.com/article/10.3390/foods13203341/s1, Figure S1: GC-MS chromatogram of bitter melon juice. (A) Fresh; (B) NFJ; (C) FJ.; Figure S2: BPI plot of positive (A) and negative (B) ions of FBMS.; Figure S3: BPI plot of positive (A) and negative (B) ions of NFBMS.; Figure S4: BPI plot of positive (A) and negative (B) ions of FJBMS.
