# Supplementary material for: Influences of Lactiplantibacillus plantarum dy-1 Fermentation on the Bitterness of Bitter Melon Juice, the Composition of Saponin Compounds, and Their Bioactivities
Source: Foods. 2024 Oct 21;13(20):3341. doi: 10.3390/foods13203341 (PMC11507596; doi:10.3390/foods13203341)
Supplement: Supplementary file 1 [file foods-13-03341-s001.zip › foods-3253706-supplementary.pdf]

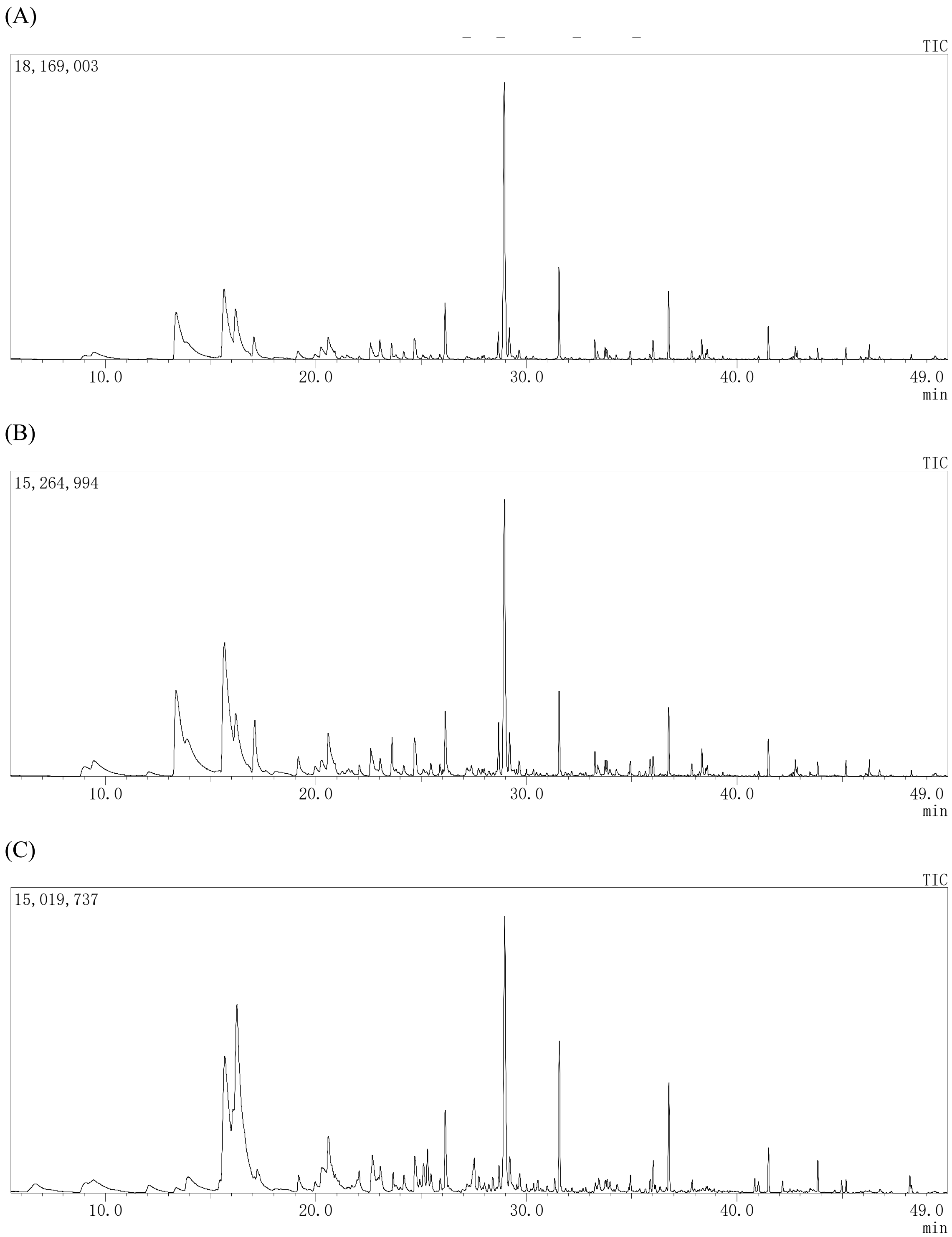

Figure S1. GCMS chromatogram of bitter melon juice. (A) Fresh; (B)NFJ; (C)FJ.

(A)

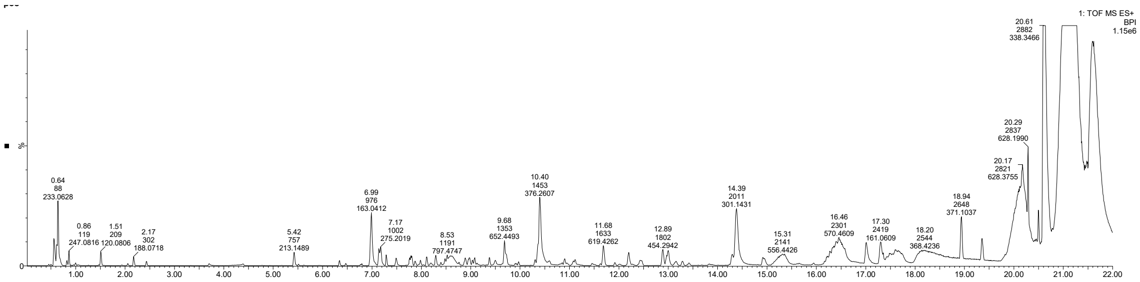

(B)

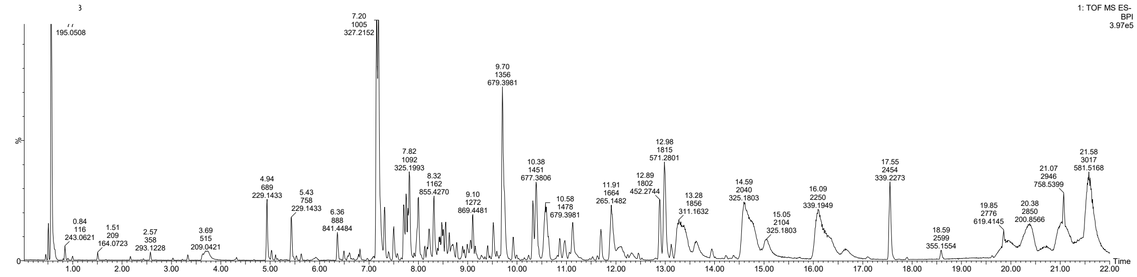

Figure S2. BPI plot of positive (A) and negative (B) ions of FBMS.

(A)

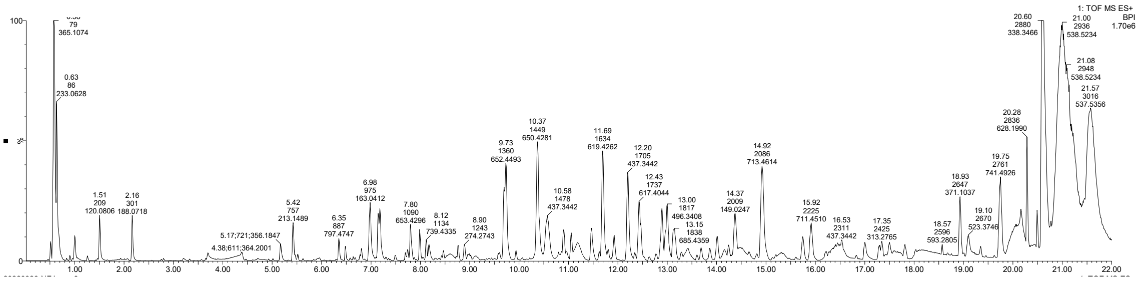

(B)

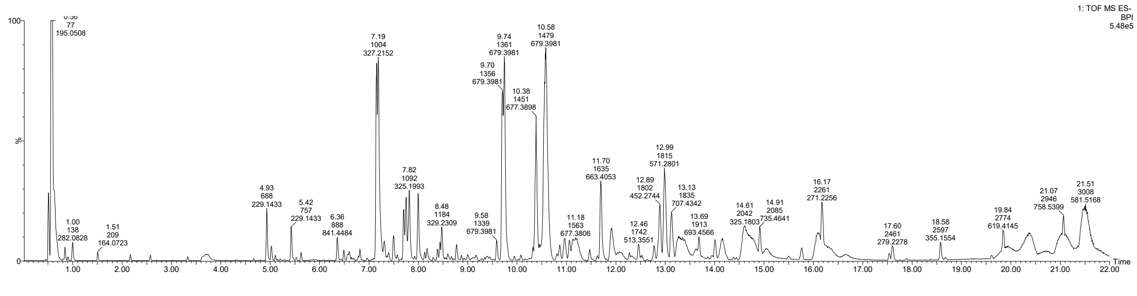

Figure S3. BPI plot of positive (A) and negative (B) ions of NFBMS.

(A)

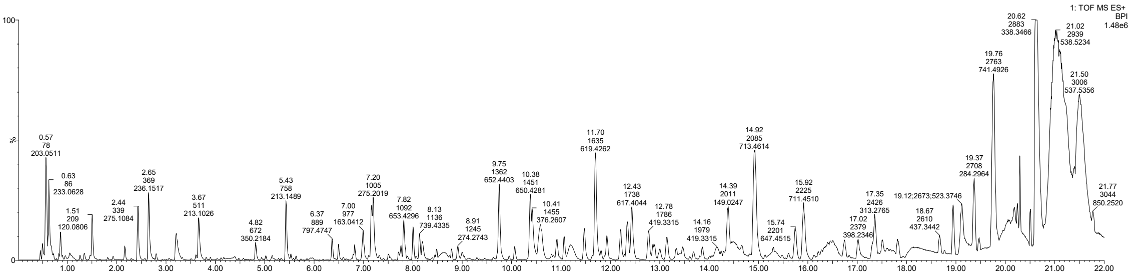

(B)

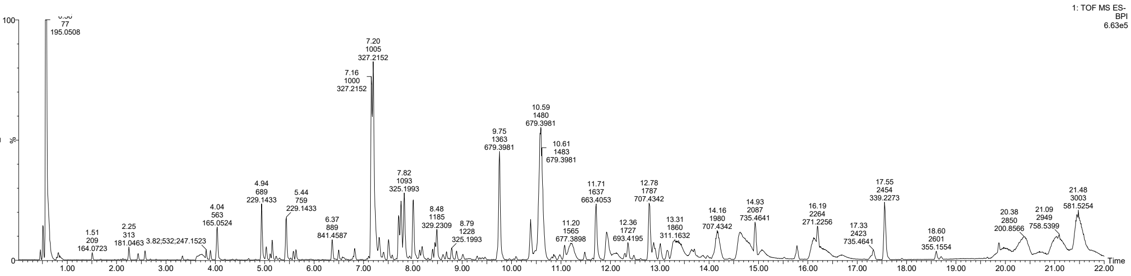

Figure S4. BPI plot of positive (A) and negative (B) ions of FJBMS.
